# The Roles of MicroRNAs in the Regulation of Rice–Pathogen Interactions

**DOI:** 10.3390/plants14010136

**Published:** 2025-01-06

**Authors:** Yanfeng Jia, Kai Wei, Jiawang Qin, Wenxue Zhai, Quanlin Li, Yalan Li

**Affiliations:** 1 Xinjiang Key Laboratory of Biological Resources and Genetic Engineering, College of Life Science and Technology, Xinjiang University, Urumqi 830046, China; yfjia@xju.edu.cn (Y.J.); kaiwei@xju.edu.cn (K.W.); qin2465606043@163.com (J.Q.); 2 Institute of Genetics and Developmental Biology, Chinese Academy of Sciences, Beijing 100101, China; wxzhai@genetics.ac.cn; 3 College of Forestry and Landscape Architecture, Xinjiang Agricultural University, Urumqi 830052, China

**Keywords:** rice, microRNAs, *Magnaporthe oryzae*, *Xanthomonas oryzae* pv. *oryzae*, *Rhizoctonia solani*

## Abstract

Rice is exposed to attacks by the three most destructive pathogens, *Magnaporthe oryzae* (*M. oryzae*), *Xanthomonas oryzae* pv. *oryzae* (*Xoo*), and *Rhizoctonia solani* (*R. solani*), which cause substantial yield losses and severely threaten food security. To cope with pathogenic infections, rice has evolved diverse molecular mechanisms to respond to a wide range of pathogens. Among these strategies, plant microRNAs (miRNAs), endogenous single-stranded short non-coding RNA molecules, have emerged as promising candidates in coordinating plant–pathogen interactions. MiRNAs can modulate target gene expression at the post-transcriptional level through mRNA cleavage and/or translational inhibition. In rare instances, they also influence gene expression at the transcriptional level through DNA methylation. In recent years, substantial advancements have been achieved in the investigation of microRNA-mediated molecular mechanisms in rice immunity. Therefore, we attempt to summarize the current advances of immune signaling mechanisms in rice–pathogen interactions that are regulated by osa-miRNAs, including their functions and molecular mechanisms. We also focus on recent findings concerning the role of osa-miRNAs that respond to *M. oryzae*, *Xoo*, and *R. solani*, respectively. These insights enhance our understanding of how the mechanisms of osa-miRNAs mediate rice immunity and may facilitate the development of improved strategies for breeding pathogen-resistant rice varieties.

## 1. Introduction

MicroRNAs (miRNAs) are endogenous, single-stranded, and small non-coding RNA molecules consisting of 21 to 24 nucleotides (nt). These molecules are distributed across the genomes of animals, plants, and microorganisms, where they interact with partially complementary sequences in target messenger RNAs (mRNAs) [1,2]. Plant miRNAs can post-transcriptionally regulate target gene expression through mRNA cleavage and/or translational inhibition [3]. In addition to these canonical miRNAs, a class of 24 nt miRNAs, referred to as long miRNAs (lmiRNAs), has been identified in rice, which directs DNA methylation to target genes to transcriptionally regulate their expression [4,5]. Over the past few years, numerous studies have demonstrated that miRNAs play broad roles in almost all various processes related to growth, development, and immunity in plants [6,7,8,9,10,11]. The biogenesis of plant miRNAs involves a series of transcription, processing, and modification processes [12]. Specifically, miRNA genes (MIRs) are transcribed into primary miRNAs (pri-miRNAs) with hundreds of nt by RNA polymerase II (Pol II). Then, the pri-miRNAs are processed sequentially into precursor miRNAs (pre-miRNAs) and short miRNA/miRNA* duplexes by the RNase III family enzyme Dicer-like1 (DCL1), a homolog of Dicer, within the nucleus. Although most miRNAs are 21 nt in length [13,14], the 22 nt isoforms of miRNAs are often produced due to the alternative or imprecise processing of pri-miRNAs by DCL1 [15]. In addition to DCL1, DCL3 can also process pri-miRNAs, resulting in the production of a class of 24 nt-long miRNAs in rice [3,4]. Subsequently, the 3’ end of the miRNA/miRNA* duplex are methylated by HUA ENHANCER 1 (HEN1) methyltransferase and are subsequently transported from the nucleus to the cytoplasm with the assistance of HASTY (HST) transporters [16]. The degradation of miRNA* strands leads to the maturation of miRNAs, which include 21 nt and 24 nt variants. These miRNAs are subsequently incorporated into the cytosolic ARGONAUTE1 (AGO1) and AGO4 proteins to form the RNA-induced silencing complex (RISC) [17]. The miRNA-AGO1 RISC mediates sequence-complementary transcript cleavage or translational repression, whereas the miRNA-AGO4 miRISC mediates target site methylation [4,18]. In addition to miRNAs, plants also contain a significant class of small RNA types known as small interfering RNAs (siRNAs). Predominantly, these endogenous siRNAs originate from the genomic repetitive sequences and intergenic regions of transposable elements (TEs), with a characteristic length of 24 nt [19]. The biogenesis of siRNAs involves their generation from perfectly complementary, long, double-stranded RNAs (dsRNAs) and subsequent processing by DCL3. These siRNAs play a crucial role in determining the specificity of the RNA-directed DNA methylation (RdDM) pathway [20].

Rice (*Oryzae sativa* L.) is one of the most momentous food crops in the world, providing approximately 21% of calories needed in humans’ daily diets [21]. Ensuring the safety of rice production has become a critical priority for human survival. Nevertheless, rice is susceptible to various survival challenges posed by pathogens from external environments, including fungi, bacteria, and viruses, throughout its lifecycle [22]. The three most destructive rice diseases are rice blast, bacterial blight (BB), and sheath blight, which are caused by the hemibiotrophic fungus *Magnaporthe oryzae* (*M. oryzae*), the hemibiotrophic Gram-negative bacterium *Xanthomonas oryzae* pv. *oryzae* (*Xoo*), and the necrotrophic fungus *Rhizoctonia solani* (*R. solani*), respectively [23,24,25]. These diseases result in severe yield losses, with annual losses attributed to pests and diseases worldwide reaching up to 30% of total rice yield [26,27], which has a severe impact on rice production and threatens human food security.

The breeding of disease-resistant varieties is the most cost-effective, eco-friendly strategy for crop disease prevention and control in agriculture. This strategy mitigates the risks associated with serious environmental pollution and food safety arising from the extensive use of chemical pesticides, as well as the challenges posed by pesticide resistance in pathogens. The successful implementation of this strategy is contingent upon the identification and cloning of *resistant* (*R)* genes, as well as the elucidation of mechanisms underlying resistance [28,29,30]. As one of the most widely studied crops, more than 50 *R* genes conferring resistance to multiple types of pathogens have been functionally identified in rice [29,31]. However, it is noteworthy that most of the identified resistance genes exhibit narrow resistance spectra, challenges in breeding, the loss of resistance over time, and high associated costs, which limits the repertoire of genes utilized in the genetic improvement of rice. Consequently, the discovery of genetic modulators that enable precise and targeted regulation, along with an understanding of the immune regulatory mechanisms in rice, is essential for the advancement of breeding strategies aimed at enhancing disease resistance [32,33]. Recent studies have uncovered a previously unrecognized layer of gene expression regulation exerted by osa-miRNAs, particularly concerning the regulation of disease-resistant genes.

**Figure 1:**
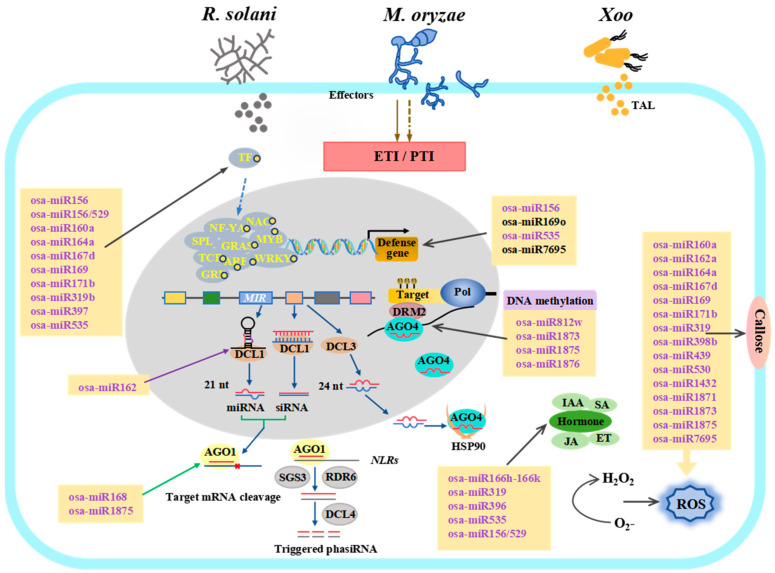
Functional osa-miRNAs related to the immune response against *M. oryzae*, *Xoo*, and *R. solani*. The identified osa-miRNAs are shown to directly or indirectly activate various biological processes related to PTI and ETI. These processes include the accumulation of ROS, callose deposition, the expression of defense-related genes, the regulation of plant hormones, and the modulation of DNA methylation, all of which contribute to the regulation of rice immunity. Solid arrows indicate direct regulation of immune signaling components and their downstream responses, while dashed arrows signify indirect or unknown regulatory mechanisms.

**Figure 2:**
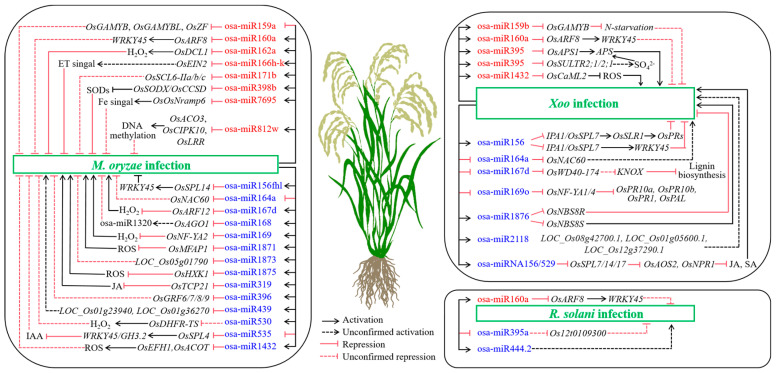
Summary of known osa-miRNA-mediated defense mechanisms in rice against *M. oryzae*, *Xoo*, and *R. solani*. Osa-miRNAs highlighted in red and blue represent positively and negatively regulated miRNAs, respectively.

Over the past few years, numerous studies have demonstrated that osa-miRNAs play broad roles in various intracellular processes related to plant growth and development, as well as in responses to abiotic and biotic stresses [6,8,34,35]. Several recent reviews have addressed the biogenesis, functions, and immune responses of osa-miRNAs in rice for readers seeking more comprehensive information [3,6,36,37,38,39]. This review aims to summarize the functional characterization and molecular mechanisms of osa-miRNAs, which act in response to *M. oryzae*, *Xoo*, and *R. solani* (Figure 1 and Figure 2, Table 1).

## 2. Functionally Characterized Osa-miRNAs Associated with the Immune Response Against *M. oryzae*, *Xoo*, and *R. solani*

Emerging evidence from a variety of experimental systems and methodologies indicates that osa-miRNA-mediated regulatory mechanisms are critical in defense-related responses to *M. oryzae*, *Xoo*, and *R. solani*. However, the complex and diverse regulatory properties of osa-miRNA present significant challenges in elucidating their roles in rice disease resistance. Researchers have demonstrated that a single mature miRNA can be transcribed from one or more MIR loci, while distinct loci may exhibit variability. Furthermore, one mature miRNA can inhibit the expression of one or several target genes, with the degree of inhibition potentially differing among various targets. Additionally, a single target gene may be regulated by multiple miRNAs [39]. The primary challenge lies in the identification of authentic targets in rice that contribute to osa-miRNA-mediated resistant or susceptible phenotypes. Plant miRNAs primarily regulate the expression of target genes through mechanisms such as mRNA cleavage, translational inhibition, or DNA methylation. Initially, targets can be predicted based on sequence complementarity using online resources such as psRNATarget [75] or psRobot [76]. For mRNA cleavage, targets can be relatively easily predicted and experimentally validated through the degradome sequencing and/or RNA ligase-mediated 5′-rapid amplification of cDNA ends (RLM-5′-RACE) [18,77]. In the case of translational inhibition, target genes can be confirmed using a reporter assay that fuses the predicted target site to a fluorescent protein [40]. For DNA methylation, target genes can be identified through bisulfite sequencing analysis [4]. Ultimately, the authenticity of target genes can be further validated through multiple omics analyses, which involve comparing the expression patterns of osa-miRNAs in transgenic lines that overexpress osa-miRNAs and their target mimics, as well as employing the CRISPR/Cas9-mediated mutagenesis of osa-miRNA loci and osa-miRNA-binding sites [78,79,80]. The critical question remains whether the identified authentic target genes contribute to the phenotypes mediated by osa-miRNAs. While the regulatory effects of most target genes of miRNAs in response to *M. oryzae* and *Xoo* have been confirmed, the identification of target genes responsive to *R. solani* is still an area requiring further investigation (Table 1) [39,81].

Similarly to *Arabidopsis*, rice possesses a two-tiered innate immune system to defend against *M. oryzae*, including pathogen-associated molecular pattern-triggered immunity (PTI) and effector-triggered immunity (ETI) [82]. These two immune responses are mediated by pattern-recognition receptors (PRRs) and nucleotide-binding leucine-rich repeat *R* proteins, respectively [83,84]. In the interactions between rice and *M. oryzae*, *Xoo*, and *R. solani*, osa-miRNAs are implicated in both PTI and ETI responses. These responses encompass various processes, including the production of reactive oxygen species (ROS), callose deposition, the reprogramming of defense-related genes, alterations in plant hormone biosynthesis and signaling, and variations in DNA methylation (Figure 1).

Numerous osa-miRNAs are known to target transcription factors, thereby regulating the transcription of defense-related genes in rice. Specifically, osa-miR167d, osa-miR169, osa-miR171b, osa-miR319b, and osa-miR535 each target distinct transcription factors, including *OsARF12*, *OsNF-YA*, *OsSCL6-IIa/b/c*, *TEOSINTE BRANCHED/CYCLOIDEA/PROLIFERATING CELL FACTOR21* (*OsTCP21*), and *OsSPL4*, to modulate rice immunity against *M. oryzae* either negatively or positively [40,41,42,43,44]. Furthermore, osa-miR160a and osa-miR164a inhibit their respective target transcription factors, *OsNAC60* and *OsARF8*, which subsequently leads to increased ROS production, callose accumulation, and the expression of defense-related genes, thereby enhancing rice immunity [45,47]. Additionally, osa-miR156, osa-miR156/529, and osa-miR169o negatively regulate rice resistance to bacterial blight by targeting *OsSPL7/14/17* and *OsNF-YA1/4*, respectively [65,66,67].

Certain osa-miRNAs are involved in the induction of *OsPR* gene expression, a phenomenon observed in resistant rice cultivars. Specifically, osa-miR156, osa-miR169o, osa-miR535, and osa-miR7695 target distinct genes, including *OsSPL7/14*, *NF-YA1/4*, *OsSPL4*, and the iron transporter gene *OsNramp6*. This targeting subsequently enhances the expression levels of defense-related genes, which encompass *OsPR* genes, *OsWRKY45*, and genes involved in diterpenoid biosynthesis [44,46,48,50,65,67].

Several osa-miRNAs mediate rice disease resistance by modulating the biosynthesis and signaling pathways of various plant hormones, including indole-3-acetic acid (IAA), ethylene (Et), jasmonic acid (JA), and salicylic acid (SA). This modulation enables rice to adapt dynamically and flexibly to a range of environmental conditions. Specifically, osa-miR166k-166h has been shown to positively regulate rice immunity against the blast fungus by suppressing *OsEIN2*, which in turn influences Et signaling [53]. Conversely, the overexpression of osa-miR319b results in the increased susceptibility of rice to blast disease by targeting *OsTCP21*, thereby promoting the expression of *OsLOX2*, *OsLOX5*, *OsCOI1*, and *OsCOI2*, which are key enzymes involved in JA biosynthesis [43,85]. Additionally, osa-miR396 and osa-miR535 negatively regulate resistance to rice blast disease by suppressing multiple *OsGRFs* and *OsSPL4*, which affects IAA homeostasis [44,54]. Furthermore, osa-miR156/529 negatively impacts rice resistance to *Xoo* by targeting *OsSPL7*, *OsSPL14*, and *OsSPL17*, which activate the transcription of *OsAOS2* and *OsNPR1*, leading to a reduction in JA content and compromised SA signaling [66].

The genes that are key components involved in the biosynthetic pathways of osa-miRNAs also play a significant role in modulating the immune response in rice. In particular, the silencing of *OsDCL1*, which is essential for the formation of mature osa-miRNAs, has been shown to enhance resistance to *M. oryzae* infection. This response parallels the effects observed with the overexpression of osa-miR162a [55]. Furthermore, osa-miR168 targets *OsAGO1*, a principal component of the RNA-induced silencing complex, and regulates the expression of osa-miR1320 and osa-miR164, thereby augmenting immunity against the blast fungus [56]. Additionally, *OsAGO2*, a core component of the RISC, interacts with 24 nt osa-miR1875 and binds to the promoter region of *OsHXK1*, which further enhances immunity against rice blast disease [57]

Several osa-miRNAs play a crucial role in regulating rice immunity through the mechanism of DNA methylation, which modulates the expression of target genes. Specifically, the DNA methylation of the *OsACO3*, *OsCIPK10*, and *OsLRR* genes, as well as the MIR812w locus, is influenced by osa-miR812W, thereby impacting immunity to rice blast disease [58]. Previous research has demonstrated that osa-miR1873 directly induces cytosine DNA methylation in *LOC_Os05g01790* and regulates its expression [4]. Further studies have indicated that osa-miR1873 fine-tunes rice immunity against *Magnaporthe oryzae* and influences yield traits through *LOC_Os05g01790* [59]. Furthermore, the *OsAGO2*/osa-miR1875 module directly interacts with the chromatin of *OsHXK1*, leading to the silencing of *OsHXK1* expression via DNA methylation, which in turn regulates rice immunity against *M. oryzae* [57]. Additionally, the resistance of *OsNBS8R* to bacterial blight is modulated by osa-miR1876 through DNA methylation, particularly in CHG sites, according to Jiang et al., 2020 [68].

## 3. Identification of Osa-miRNAs Involved in Response to *M. oryzae* in Rice

Rice blast, caused by the fungal pathogen *M. oryzae*, is a geographically widespread and devastating disease that seriously affects both the yield and quality of rice [86]. Understanding and analyzing the resistance mechanisms associated with this disease can enhance disease prevention and control strategies, and numerous breakthroughs have been achieved in recent decades. To date, more than 100 rice blast *R* genes have been identified within the rice genome, of which 30 genes, encoded by *nucleotide-binding site leucine-rich repeat* (*NLR*) family receptors, have been functionally characterized [87,88,89]. Additionally, at least 11 avirulence effectors from *M. oryzae* have been identified [90]. Previous studies have shown that a large proportion of osa-miRNAs regulate the progression of *M. oryzae* infection and/or the response to the *M. oryzae* elicitor in rice plants [37,39,46,51,91,92,93]. These osa-miRNAs are predicted to regulate multiple signaling pathways by targeting a series of genes, including those involved in the biogenesis of small RNAs and defense pathways, as well as IAA, SA, JA, ET signaling, and ROS signaling [37,43,47,49,57]. In addition, they can also target some genes containing upstream open reading frames (ORF) to regulate these signaling pathways [44]. The identification of these osa-miRNAs opens up new avenues to explore the regulatory mechanisms underlying rice immunity against *M. oryzae*.

### 3.1. The Osa-miRNAs That Play Positive Roles in Rice Immunity Against M. oryzae

In previous studies, at least eight osa-miRNAs have been reported to act as positive regulators in the interaction between rice and *M. oryzae* by suppressing their respective target genes, including osa-miR7695 [50,51,52], osa-miR160a [45,46], osa-miR166h-166k [53], osa-miR398b [46,49], osa-miR162a [55], osa-miR159a [60], osa-miR812w [58], and osa-miR171b [42].

Osa-miR159 is a conserved osa-miRNA that generates five mature isoforms, namely osa-miR159a/b/c/d/e/f. This osa-miRNA has been revealed to mediate the cleavage of mRNA for three target genes, *OsGAMYB*, *OsGAMYBL*, and *OsZF* [94,95]. Several studies have shown the important role of miR159 in the immune responses of various plant species, such as cotton [96], lily [97], tomato [98], and apple [99]. Notably, overexpressing osa-miR159a in transgenic plants alleviated rice’s susceptibility to *M. oryzae* infection. In contrast, transgenic plants expressing a short-tandem target mimic (STTM) to block osa-miR159a showed susceptibility. Meanwhile, rice plants with knockout mutations of the three target genes, *OsGAMYB*, *OsGAMYBL* and *OsZF*, enhanced resistance to *M. oryzae*. In addition, alterations in the expression of osa-miR159a were also found to affect yield traits, including pollen and grain development [60].

The osa-miR812 family consists of 22 (osa-miR812a-v) members in the rice genome. Notably, osa-miR812w, a new member that was previously named as miR-75 [51], has been reported to be involved in rice blast resistance [58]. osa-miR812w is present in multiple *Oryza* cultivar species, including the *japonica* and *indica* subspecies, as well as in wild rice species osa-miRNAs [58]. However, it is absent in other monocotyledonous and dicotyledonous plant species [58]. Simultaneously, 24 nucleotide-derived osa-miR812w-3p species, processed by DCL3, have been shown to associate with AGO4 [4]. Furthermore, overexpressing osa-miR812w in transgenic plants increases resistance against infection due to the rice blast fungus *Magnaporthe oryzae*, which is attributed to the accumulation of higher levels of hydrogen peroxide (H_2_O_2_). In contrast, CRISPR/Cas9-mediated osa-miR812w editing in plants enhances disease susceptibility, characterized by reduced H_2_O_2_ levels. This process may be mediated through the expressed regulation of three target genes, *1-aminocyclopropane-1-carboxylate oxidase3* (*ACO3*), *calcineurin B-like (CBL)-interacting serine–threonine protein kinase* (*CIPK10*), and *leucine repeat-containing protein* (*LRR*) expression, and potentially through DNA methylation [58].

A previous study demonstrated that the accumulation of osa-miR171b contributed to disease resistance against rice stripe virus (RSV) infection by modulating disease symptoms [100]. This finding suggested that miR171b functions as a regulator of plant resistance. The transgenic plants overexpressing osa-miR171b displayed a marked increase in resistance to rice blast, a slight reduction in grain yield, and a delay in flowering. This was attributed to the inhibition of three *SCARECROW-Like* (*SCL*) target genes: *OsSCL6-IIa*, *OsSCL6-IIb*, and *OsSCL6-IIc*, which may interact with DELLAs to disrupt the DELLA-JAZ association and JA responses [42,101,102,103,104]. Meanwhile, the osa-miR171b-*OsSCL6-IIs* module exhibited dynamic expression throughout the rice growth stages [42]. During the vegetative stage, osa-miR171b was up-regulated to suppress the expression of *OsSCL6-IIs*, which improved the disease resistance. Conversely, osa-miR171b was down-regulated during the reproductive stage while *OsSCL6-IIs* was up-regulated, facilitating normal development and the flowering of panicles [42].

### 3.2. The Osa-miRNAs That Facilitate M. oryzae Attack in Rice

Currently, at least 13 osa-miRNAs have been found to facilitate *M. oryzae* attack in rice, including osa-miR156 [48], osa-miR164a [47], osa-miR167d [41], osa-miR168 [56], osa-miR169 [40], osa-miR1871 [61], osa-miR1873 [59], osa-miR1875 [57], osa-miR319 [43], osa-miR396 [54], osa-miR439 [62], osa-miR530 [63], osa-miR535 [44], and osa-miR1432 [64].

Osa-miR1871 is a rice-specific 24 nucleotide osa-miRNA that responds to both abiotic and biotic stresses [46,105]. Transgenic plants with overexpressed osa-miR1871 demonstrated compromised resistance to blast disease and a reduction in grain yield. In contrast, the inhibition of osa-miR1871 via a target mimic (MIM1871) resulted in enhanced immunity and yield when compared to the Nipponbare control [61]. In addition, blocking osa-miR1871 led to a constitutive increase in the expression of genes associated with chitin responsiveness, thereby priming PTI responses [61]. The *microfibrillar-associated protein* (*MFAP1)*, a growth-related marker gene in the fungus *Cenangium ferruginosum* [106], is the target gene of osa-miR1871 in rice [61]. Notably, MFAP1 is located near the cell wall and is found to positively regulate PTI responses with higher ROS levels and more callose deposits [61]. The phenotypic characteristics of the *MFAP1* mutants were similar to those of the transgenic plants overexpressing osa-miR1871. Conversely, transgenic lines overexpressing MFAP1 or both MFAP1 and osa-miR1871 exhibited comparable resistance to MIM1871 [61]. Furthermore, time-course experimental data indicated that the expression levels of osa-miR1871 and *MFAP1* in rice leaves, panicles, and basal internodes exhibited dynamic changes throughout the entire growth period, thereby playing a crucial role in the regulation of immune responses and yield traits [61].

Mature sequences of osa-miR535 exhibit a high similarity to osa-miR156 and osa-miR529, despite their derivation from distinct MIR genes [107]. These osa-miRNAs target the same transcription factor gene family, specifically the *SQUAMOSA (SQUA) promoter-binding-like* (*SPL*) family, which comprises 19 members in rice and plays a crucial role for the regulation of plant growth and immunity [48,107,108,109,110,111,112]. Osa-miR156 has been verified as a negative regulator of rice immunity against *M. oryzae* infection [48]. Transgenic plants with overexpressed osa-miR156 enhance their susceptibility to blast disease, whereas blocking osa-miR156fhl-3p via a target mimic of osa-miR156 (MIM156) confers higher resistance to *M. oryzae* [48]. Furthermore, osa-miR156 can specifically combine with the *OsSPL14* gene, which has been reported to enhance rice resistance against *M. oryzae* and inhibit the expression of *OsSPL14* and *WRKY45*, thereby compromising blast resistance [48,113]. Concurrently, osa-miR535 targets another *SPL* gene, *OsSPL4*, to negatively regulate rice immune responses to *M. oryzae* infection. Consistently, transgenic rice lines overexpressing *OsSPL4* increase blast disease resistance and delay *M. oryzae* infection [44]. *OsSPL4* also binds to the promoter of *GH3.2*, an indole-3-acetic acid-amido synthetase, which affects IAA homeostasis and promotes its expression. This disruption of IAA homeostasis results in a decrease in free IAA levels, ultimately causing the suppression of the IAA signaling pathway and enhancing rice blast resistance [44].

ARGONAUTE proteins (AGOs) are essential components of RISCs and are involved in the regulation of sRNA to regulate rice immune responses [56,57,114,115,116]. Previous research has illustrated that *OsAGO2* can improve rice’s sensitivity to rice black-streaked dwarf virus infection via controlling the DNA methylation of *HEXOKINASE1* (*OsHXK1*) [116]. *HXK1* can transmit glucose signals as a glucose sensor [117] and regulate plant immunity to pathogens by mediating ROS, anthocyanin accumulation, and stomatal closure [116,118,119,120,121]. *OsAGO2* can also negatively regulate resistance to blast disease by binding to 24 nucleotide osa-miR1875, which mediates DNA methylation and leads to the silencing of *OsHXK1* [57]. Conversely, *OsHXK1* acts as a positive regulator of resistance to blast disease, likely through its role as a glucose sensor, which induces the expression of defense-related genes and promotes the accumulation of ROS [57].

Additionally, osa-miR160a [45,46], osa-miR162 [55], osa-miR168 [56], osa-miR171b [42], osa-miR1871 [61], osa-miR1873 [59], osa-miR396 [54], osa-miR530 [63], and osa-miR1432 [64] have been shown to balance rice growth, development, and defense against *M. oryzae*. This balance is essential for practical applications aimed at enhancing rice immunity and yield.

## 4. Identification of RiceOsa-miRNAs Responsive to *Xoo*

Rice bacterial blight (BB) is one of the most deadly bacterial diseases, resulting in yield losses of 20–30%. It is primarily caused by the Gram-negative proteobacterium *Xoo* [122,123] and serves as an exemplary pathosystem for characterizing the interactions between plant hosts and bacterial pathogens [124].

Previous studies have identified at least 16 protein-coding genes that confer either dominant or recessive resistance to *Xoo* in rice [125,126,127,128,129,130,131,132,133,134,135,136,137,138,139,140,141]. The utilization of rice germplasm resources that harbor these resistance genes represents a highly effective, economical, and environmentally sustainable strategy for the prevention and control of plant diseases in agricultural settings [33]. However, the effectiveness of this approach is often compromised by the loss of resistance and the emergence of new virulent pathogen populations over time [142,143,144,145,146,147]. An alternative strategy involves the reprogramming of the expression patterns of TALE-triggered susceptibility genes, which can be achieved through genome-wide association studies (GWAS) [148,149] and Cas9-mediated gene editing techniques [150,151,152,153,154]. In addition to the GWAS and TALE-dependent resistance gene mining methods, the exploration of miRNA-targeted genes may offer a promising avenue for the discovery of resistance-associated genes.

Although certain osa-miRNAs have been characterized for their role in regulating disease resistance, the specific functions of miRNAs in the interaction between rice and *Xoo* remain relatively unknown. A number of osa-miRNAs and their corresponding target genes that respond to *Xoo* have been identified through the analysis of dynamic expression level changes in the various rice varieties using small RNA sequencing, transcriptome analysis, and degradome sequencing [69,155,156,157].

### 4.1. The Osa-miRNAs That Play Positive Roles in Rice Immunity Against Xoo

To date, at least four osa-miRNAs have been proposed to positively regulate the interaction between rice and *Xoo* by suppressing their target genes, including osa-miR159b [69], osa-miR1432 [70], osa-miR160a [45], and osa-miR395 [71].

Osa-miR395 is a differentially expressed osa-miRNA that is activated following inoculation with *Xoo*, distinguishing the rice susceptible line Mudanjiang 8 (MDJ8) from the resistant line Rb49, which harbors the major disease resistance gene *Xa3/Xa26* [156].

Osa-miR395 currently comprises 25 members within the rice genome, which generally demonstrate high expression levels and conservation across species [158]. It enhances resistance against two destructive bacterial pathogens, *Xoo* and *X. oryzae pv. Oryzicola* (*Xoc*), by modulating PTI responses and R (*Xa3*/*Xa26* and *Xa23*) gene-mediated resistance [71]. Utilizing the psRNATarget and WMD3 bioinformatics tools, *OsAPS1*, which functions in sulfate assimilation, along with two sulfate transporter genes, *OsSULTR2;1* and *OsSULTR2;2*, known for their sulfate transport activity that facilitates sulfate accumulation, have been predicted as targets of osa-miR395 [159,160].

Moreover, osa-miR395 can disrupt sulfate translocation in the rice xylem by inhibiting the expression of *OsAPS1*, *OsSULTR2;1*, and *OsSULTR2;2* [71]. The regulation of sulfate accumulation by osa-miR395, rather than S-metabolites, impairs the virulence of *Xoo* and *Xoc*, consequently preventing their proliferation and resulting in broad-spectrum resistance to both pathogens [71].

### 4.2. The Osa-miRNAs That Facilitate Xoo Attack in Rice

Currently, a minimum of seven osa-miRNAs have been identified as facilitating the attack of *Xoo* in rice, including osa-miR169o [67], osa-miR156 [65], osa-miR164a [69], osa-miR167d [69], osa-miR1876 [68], osa-miR2118 [72], and osa-miRNA156/529 [66].

Osa-miR169 is a highly conserved osa-miRNA in *Oryza sativa*, encoded by 18 family members, which include osa-miR169a through to osa-miR169r (http://www.mirbase.org/). Several of these members have been shown to be regulated in response to abiotic stresses in various plant species, such as *Arabidopsis*, maize, and soybean, under drought stress [161], N deficiency [162], low temperatures [163] and salt tolerance [164]. Furthermore, the target of osa-miR169, which encodes *subunit A of the nuclear factor Y* (*NF-YA*), also responds to abiotic stresses in rice [165,166,167,168,169]. Recent studies have indicated that 10 subunits of osa-miR169, specifically osa-miR169o/n and osa-miR169f.1/g/h/i/j/k/l/m, are down-regulated under *Xoo* inoculation, nitrogen-limiting stress, and combined stress in rice [67]. Eight of the eleven *NF-YA* genes have been identified as the authentic targets of osa-miR169, including from *NF-YA1* to *NF-YA6*, as well as *NF-YA10* and *NF-YA11* [18,40,170,171,172]. The overexpression of osa-miR169o in transgenic plants has been shown to weaken rice immunity to *Xoo* infection, N limitation stress, and combined stress by suppressing the expression of *NF-YA1* and *NF-YA4* [67]. Consistently, the transient expression of *NF-YA4*, *NF-YA10*, and *NF-YA11* in rice protoplasts result in a significant up-regulation of *pathogenesis-related* (*PR)* genes and *NRT2* genes, while slightly reducing the transcriptional activity of genes in the *NRT1* family [67].

Osa-miR156 is a highly expressed and conserved osa-miRNA in the rice genome, comprising 12 distinct members. This osa-miRNA targets 11 *SQUAMOSA-PROMOTER BINDING PROTEIN-LIKE* (*SPL)* genes in rice, which are involved in regulating significant agronomic traits [108]. *SPL14*, also known as *ideal plant architecture 1* (*IPA1*), can enhance rice immunity through a phosphorylated process that alters its DNA binding specificity and activates the expression of *OsWRKY45* in response to blast inoculation [113]. Additionally, osa-miR156 negatively regulates resistance against *Xoo* based on the physical interaction between target genes (*IPA1* and *OsSPL7*) and the DELLA protein SLENDER RICE 1 (SLR1). This process enhances the stability of SLR1 to reduce gibberellin acid (GA)-mediated susceptibility to *Xoo* [65]. Further investigations reveal that *Xoo* infection simultaneously activates the transcription of miR156 and miR529 while suppressing the expression of three target genes, *OsSPL7*, *OsSPL14*, and *OsSPL17* [66]. The proteins OsSPL7, OsSPL14, and OsSPL17 directly bind to the promoter regions of their downstream target genes, rice *allene oxide synthase 2 (OsAOS2)* and *NONEXPRESSOR OF PATHOGENESIS-RELATED GENES1 (OsNPR1)*, thereby activating their expression. This regulatory mechanism influences JA accumulation and the SA signaling pathway, facilitating *Xoo* infection [66]. Non-TAL effector XopQ-inducible osa-miR1876 was initially identified as a regulator of the *OsNBS8R* gene through DNA methylation, which encodes an NB-ARC protein and confers quantitative resistance to *Xoo* [68]. The binding site of osa-miR1876 is located in the 5′UTR of *OsNBS8R*, inserted randomly, and has evolved alongside osa-miR1876. The interaction between *OsNBS8R* and XOPQ-induced osa-miR1876 partially aligns with the zigzag model, suggesting that *OsNBS8R* may also adhere to this framework to regulate innate immune responses or basal disease resistance aganist *Xoo* [68].

In addition, osa-miR156 [65] and osa-miR169o [67] have been reported to improve yield, nitrogen use’s efficiency, and disease resistance to *Xoo*, respectively. This could establish a new strategy for achieving both high disease resistance and high yield for rice breeding.

## 5. Identification of Rice Osa-miRNAs Responsive to *R. solani*

Rice sheath blight (RSB) is a major fungal disease caused by the soil-borne necrotrophic fungus *R. solani* AG1-IA, which leads to substantial yield losses and a decline in grain quality [23,173,174]. Resistance to RSB is a highly complex quantitative trait characterized by dynamic and diverse responses that are regulated by multiple genes [175]. The dominant resistance genes or loci associated with RSB have not been identified in rice, indicating that the mechanisms underlying rice resistance to *R. solani* AG1-IA remain largely unexplored [176,177]. Emerging evidence indicates that osa-miRNAs play a pivotal role in host–fungal interactions and resistance responses. However, the majority of existing studies have concentrated on *M. oryzae*, a hemibiotrophic fungus [178]. Consequently, there is a notable lack of research regarding the genetic regulation of miRNA-mediated interactions between rice and *R. solani*.

Consistent with the ongoing research described above, there is only limited knowledge about the details of *R. solani*-responsive miRNAs [177]. Five independent studies have examined the expression profiles of osa-miRNAs across various rice cultivars during *R. solani* infection, utilizing high-throughput small RNA sequencing methodologies [73,178,179,180,181]. Although the mechanisms of rice–*R. solani* interactions regulated by osa-miRNAs are emerging, only three osa-miRNAs, osa-miR160a [45], osa-miR395a [73], and osa-miR444.2 [74], have been identified as involved in the regulation of rice sheath blight.

In rice, three mature osa-miR160 variants are distinguished by a single nucleotide substitution and target multiple *AUXIN RESPONSE FACTOR* (*ARF*) genes [18,46,182]. The osa-miR160a-*ARFs* module has been reported to play a role in the basal defense response of *Arabidopsis* [183] and in resistance to rice blast disease in the *indica* cultivar Kasalath [46]. Furthermore, osa-miR160a has been shown to enhance broad-spectrum disease resistance in rice against the pathogens *M. oryzae*, *Xoo*, and *R. solani* by downregulating the expression of a subset of *ARF* genes [45]. Among these ARFs, ARF8 serves as a negative regulator of broad-spectrum disease resistance by directly binding to the promoter region of *WRKY45* [45]. These findings offer new resources to potentially improve disease resistance in rice breeding.

Through a high-throughput small RNA sequencing analysis of the indica rice variety Pusa Basmati-1 infected by *R. solani* at various time points, it was observed that osa-miR395a, osa-miR408-3p, osa-miR171i-5p, and osa-miR1861d, along with a novel osa-miRNA, osa-NmiR1, exhibited responsiveness to the dynamic and coordinated transcriptional changes induced by *R. solani* [73]. Osa-miR395a was found to bind to and cleave the mRNA coding for pentatricopeptide repeat (PPR) proteins [73]. In alignment with previous studies, the pentapeptide (*Os12t0109300-01*; *LOC-Os12g01850*) was identified as a high-confidence target for rice osa-miR395a [184]. In rice, *OsPPRs*, specifically *OsPPR6*, have been implicated in the processes of chloroplast RNA editing and processing [185], which are facilitated by the E and DYW domains located at the C-terminus [186,187,188,189,190,191,192,193]. Furthermore, the increased expression of the *pentatricopeptide repeat for germination on NaCl* (*PGN*) gene has been reported in response to necrotrophic fungal pathogens, *Alternaria Brassicicola* (*A. brassicicola*) and *Botrytis cinerea* (*B. cinerea*), *in Arabidopsis* [194]. Consistent with the findings of [194], infection by *R. solani* resulted in the up-regulation of *PPR* via osa-miR395a, potentially due to the induction of chloroplast degradation [73].

Osa-miR444 comprises six members: osa-miR444a, b, c, d, e, and f. Among these, osa-miR444b and osa-miR444a differ by only one single nucleotide and have been reported to play a significant role in the response to environmental stress [195]. Osa-miR444a is involved in the regulation of the NO^3−^ signaling pathway, which is critical for nitrate-dependent root growth, nitrate accumulation, and responses to phosphate starvation. This regulation is accompanied by the suppression of *MADS-box* gene expression [18,196,197]. Furthermore, the overexpression of osa-miR444 in transgenic plants has been shown to enhance rice resistance to RSV infection by up-regulating the expression of *OsRDR1*, thereby facilitating the RNA silencing of RSV [198]. In contrast, the overexpression of osa-miR444b.2 has been associated with reduced resistance to *R. solani*, which is attributed to its impact on the expression of genes related to plant hormone signaling pathways, such as ET and IAA, as well as transcription factors including WRKYs and F-box proteins [74]. Additionally, the overexpression of osa-miR444b.2 has been found to influence agronomic traits in rice, resulting in a decrease in the number of primary branches and tillers, while simultaneously increasing panicle length [74].

## 6. Conclusions and Perspectives

In summary, significant progress is clearly being made in elucidating the role of osa-miRNAs in conferring immunity against rice blast disease and bacterial leaf blight. However, the comprehension of the interaction between osa-miRNAs and rice sheath blight remains limited. Future research should further elucidate the role of osa-miRNAs and identify novel osa-miRNAs in rice–pathogen interactions against *Xoo*, *M. oryzae*, and particularly *R. solani*. Additionally, it is imperative to explore the functions of key components within pertinent osa-miRNAs signaling pathways. It is unequivocal that the continued identification, validation, and analysis of more osa-miRNA modules, along with the advancement of osa-miRNA-based breeding strategies, will enhance the application of osa-miRNAs in improving rice disease resistance and ensuring crop security.

The goal of breeding is to produce high-yielding and disease-resistant rice varieties. This can be achieved by enhancing the expression of target genes and/or suppressing the accumulation of miRNAs or taking the opposite of the two approaches [11]. Specific strategies may include creating osa-miRNA-cleavage-resistant targets, utilizing constitutive or inducible promoters, applying STTM, and employing the CRISPR/Cas-mediated editing of osa-miRNAs and their targets [6,11].

## Figures and Tables

**Table 1:** List of osa-miRNAs responsive to *M. oryzae*, *Xoo*, and *R. solani*.

Pathogens	osa-miRNAs	Target Genes	Role	References
*M. oryzae*	osa-miR169	*OsNF-YA2*	Negative	[40]
	osa-miR167d	*OsARF12*	Negative	[41]
	osa-miR171b	*OsSCL6-IIa*, *OsSCL6-IIb*, *OsSCL6-IIc*	Positive	[42]
	osa-miR319	*OsTCP21*	Negative	[43]
	osa-miR535	*OsSPL4*	Negative	[44]
	osa-miR160a	*OsARF8*	Positive	[45,46]
	osa-miR164a	*OsNAC60*	Negative	[47]
	osa-miR156fhl	*OsSPL14*	Negative	[48]
	osa-miR398b	*OsCSD1*, *OsCSD2*, *OsSODX*, *OsCCSD*	Positive	[46] [49]
	osa-miR7695	*OsNramp6*	Positive	[50] [51] [52]
	osa-miR166h-166k	*OsEIN2*	Positive	[53]
	osa-miR396	*OsGRF6*, *OsGRF7*, *OsGRF8*, *OsGRF9*	Negative	[54]
	osa-miR162a	*OsDCL1*	Positive	[55]
	osa-miR168	*OsAGO1*	Negative	[56]
	osa-miR1875	*OsHXK1*	Negative	[57]
	osa-miR812w	*OsACO3*, *OsCIPK10*, *OsLRR*	Positive	[58]
	osa-miR1873	*LOC_Os05g01790*	Negative	[59]
	osa-miR159a	*OsGAMYB*, *OsGAMYBL*, *OsZF*	Positive	[60]
	osa-miR1871	*OsMFAP1*	Negative	[61]
	osa-miR439	*LOC_Os01g23940*, *LOC_Os01g36270*	Negative	[62]
	osa-miR530	*OsDHFR-TS*	Negative	[63]
	osa-miR1432	*OsEFH1*, *OsACOT*	Negative	[64]
*Xoo*	osa-miR160a	*OsARF8*	Positive	[45]
	osa-miR156	*OsSPL14 (IPA1)*, *OsSPL7*	Negative	[65]
	osa-miRNA156/529	*OsSPL7/14/17*	Negative	[66]
	osa-miR169o	*OsNF-YA1*, *OsNF-YA4*	Negative	[67]
	osa-miR1876	*OsNBS8R* *OsNBS8S*	Negative	[68]
	osa-miR159b	*OsGAMYB*	Positive	[69]
	osa-miR164a	*OsNAC60*	Negative	[69]
	osa-miR167d	*OsWD40-174*	Negative	[69]
	osa-miR1432	*OsCaML2*	Positive	[70]
	osa-miR395	*OsAPS1*, *OsSULTR2;1*, *OsSULTR2;2*	Positive	[71]
	osa-miR2118	*LOC_Os08g42700.1*, *LOC_Os01g05600.1*, *LOC_Os12g37290.1*	Negative	[72]
*R. solani*	osa-miR160a	*OsARF8*	Positive	[45]
	osa-miR395a	*Os12t0109300*	Negative	[73]
	osa-miR444.2	-	Negative	[74]

This list only includes the miRNAs that are functionally characterized.
